# Nitric Oxide Mediates Molybdenum-Induced Antioxidant Defense in Wheat under Drought Stress

**DOI:** 10.3389/fpls.2017.01085

**Published:** 2017-06-23

**Authors:** Songwei Wu, Chengxiao Hu, Qiling Tan, Shoujun Xu, Xuecheng Sun

**Affiliations:** ^1^Key Laboratory of Arable Land Conservation (Middle and Lower Reaches of Yangtze River), Ministry of Agriculture, Huazhong Agricultural UniversityWuhan, China; ^2^Hubei Provincial Engineering Laboratory for New-Type Fertilizers, Huazhong Agricultural UniversityWuhan, China

**Keywords:** Molybdenum, antioxidant defense, nitrate reductase, nitric oxide, drought stress, winter wheat

## Abstract

Molybdenum (Mo) has been reported to alleviate drought stress by enhancing antioxidant defense in plants, but the underlying mechanism remains unclear. Here, we hypothesized that Mo mediates nitric oxide (NO)-induced antioxidant defense through Mo-enzymes, particularly by nitrate reductase (NR) in wheat under drought stress. The 30-day-old wheat seedlings cultivated in -Mo (0 μM Mo) and +Mo (1 μM Mo) Hoagland solutions were detached and then pretreated with Mo-enzyme inhibitors, NO scavengers, NO donors or their combinations according to demands of complementary experiment under 10% polyethylene glycol 6000 (PEG)-stimulated drought stress (PSD). Mo supplementation increased the activities and transcripts of antioxidant enzymes, decreased H_2_O_2_ and MDA contents, and elevated NO production, implying that Mo-induced antioxidant defense may be related to NO signal. Complementary experiment showed that NO production was induced by Mo, while suppressed by Mo-enzyme inhibitors and NO scavengers, but restored by NO donors, suggesting that Mo-induced increase of NO production may be due to the regulation by Mo-enzymes. Further experiment indicated that the increased activities and transcripts of antioxidant enzymes induced by Mo were suppressed by Mo-enzyme inhibitors and NO scavengers, and NO donors could eliminate their suppressing effects. Moreover, Mo application increased NR activity and inhibitors of Mo-enzymes inhibited NR activity in wheat leaves under PSD, suggesting that NR might involve in the regulation of Mo-induced NO production. These results clearly indicate that NO mediates Mo-induced antioxidant defense at least partially through the regulation of NR.

## Introduction

Drought stress is a major environmental stress that limits plant growth and crop production. However, plants have developed various complicated adaptive mechanisms to cope with drought stress by triggering a series of physiological and biochemical responses (He et al., 2015). Among these mechanisms, antioxidant defense plays a crucial role in the alleviation of damage caused by drought stress (Wu et al., 2014; Wu S. et al., 2015). Phytohormones and signal molecules are involved in the regulation of antioxidant defense, and both play vital roles in the perception and transduction of signals induced by drought stress (Elisabeth et al., 2014). The signals activate antioxidant enzymes to destroy reactive oxygen species (ROS), which contributes to the maintenance of membrane integrity and thus confers drought tolerance to plants.

As a stress-resistance element, Molybdenum (Mo) has been extensively reported to facilitate the improvement of abiotic stress tolerance against salinity, low temperature and water stress in plants (Sun et al., 2009; Zhang et al., 2012; Sun X. et al., 2014; Wu et al., 2014). Among the mechanisms underlying tolerance improvement in plants, improvement of antioxidant defense by Mo is a crucial strategy for plants to adapt to abiotic stresses. However, little is known about the potential mechanism by which Mo improves antioxidant defense under abiotic stresses, particularly drought stress.

As a Mo-enzyme that catalyzes the reduction of nitrate to nitrite (Babenko et al., 2015), nitrate reductase (NR) also plays a key role in nitrogen (N) assimilation. In addition, NR is involved in the production of signal molecule nitric oxide (NO), and NR-dependent NO production increases the ability of nitrogen uptake (Sun et al., 2015). In plants, there are two main pathways to produce NO: reduction of nitrite to NO by NR and oxidization of arginine by nitric oxide synthase (NOS) (Elisabeth et al., 2014). More recently, it was suggested that the Mo-enzymes NR and amidoxime reducing component (ARC) could form a dual system of NR-ARC to induce NO production under nitrate in *Chlamydomonas* (Chamizo-Ampudia et al., 2016). NO plays crucial roles in plant growth, development and response to various environmental stresses (Sanz et al., 2015). There are growing evidences suggesting that NO interacts with abscisic acid (ABA), hydrogen peroxide (H_2_O_2_), brassinosteroid (BR) and other cellular mediators in response to abiotic stresses (Zhang et al., 2011; Lu et al., 2014; Sanz et al., 2015; Chen et al., 2016). NO improves seminal root elongation, which is involved in the regulation of strigolactone under nitrogen-deficient and phosphate-deficient conditions (Sun et al., 2016). NO in guard cells regulates K^+^ and anion fluxes by ion channels and it is required for ABA-induced stomatal closure in Arabidopsis (Chen et al., 2016). NO acts downstream signal of auxin to trigger root ferric-chelate reductase activity in response to iron deficiency in Arabidopsis (Chen et al., 2010). Besides, NO is involved in the improvement of oxidative stress tolerance by inducing antioxidant defense under aluminum (Al) stress in wheat roots (Sun C. et al., 2014), water stress in maize (Sang et al., 2008a), and heat stress in reed (Song et al., 2006). Calcium-calmodulin plays a role in both upstream and downstream signal of NO signal in ABA-induced oxidative tolerance in maize (Sang et al., 2008b). It has also been suggested that mitogen-activated protein kinase (MAPK) and calcium/calmodulin-dependent protein kinase (CCaMK) are downstream signal of NO signal in the induction of oxidative tolerance (Zhang et al., 2007; Ma et al., 2012). However, whether Mo induces the generation of NO by Mo-enzymes, particularly by NR, remains to be elucidated.

Here, we hypothesized that Mo induces the generation of NO by Mo-enzymes, particularly by NR, and then mediates the antioxidant defense in winter wheat under drought stress. Testing of this hypothesis may facilitate a better understanding of the mechanism by which Mo improves drought stress tolerance in plants.

## Materials and Methods

### Plant Materials and Growth Conditions

Seeds of winter wheat (Jing 852) (*Triticum aestivum*, L.) were sterilized with a 0.5% NaClO solution, and subsequently germinated for 5 days in deionized water at 25°C. Wheat seedlings of uniform size were transferred to plastic containers containing 10 L of one-quarter strength Hoagland solution. Small sponges were used to fix the wheat seedlings into the perforated lids of the containers. Plant growth conditions and the modified Hoagland solution have been described previously (Wu et al., 2014). The wheat seedlings were grown in 1/4 and 1/2 strength Hoagland solutions for the first and second 5-day periods, respectively, and then a full-strength Hoagland solution was used until the seedlings were 30-days-old. The solution was renewed every 5 days and was continuously aerated. Mo concentrations of 0 and 1 μM were denoted as “-Mo” and “+Mo,” respectively.

### Experimental Treatments with Complementary Chemicals

The 30-day-old seedlings were treated using the methods described in previous studies (Ding et al., 2012; Zhu et al., 2013; Zhang et al., 2014). The seedlings were detached at the base of stem and placed in distilled water for 2 h to eliminate potential wound stress. The detached seedlings were placed in beakers wrapped with aluminum foil containing 10% polyethylene glycol 6000 (PEG) (w/v) to simulate drought stress for 0, 3, 6, and 24 h. Based on the results of antioxidant enzyme activities, a 6-h period of drought stress was selected for further experiments.

To study the effects of various inhibitors or scavengers, the detached seedlings were firstly pretreated with 100 μM tungsten (W, Mo-enzymes inhibitors) (Lu et al., 2014; Sun C. et al., 2014) or 200 μM 2-phenyl-4,4,5,5-tetramethylimidazoline-3-oxide-1-oxyl (PTIO, NO scavenger) (Zhang et al., 2009; Wang et al., 2010; Lu et al., 2014) for 4 h, and then exposed to 10% PEG with 100 μM sodium nitroprusside (SNP, NO donor) (Zhang et al., 2007, 2009, 2011; Lu et al., 2014) for 6 h. The detailed experimental design was as below: -Mo; +Mo; -Mo +SNP; +Mo +PTIO; +Mo+W; +Mo+W+SNP. -Mo and +Mo treatments represented wheat grown in modified Hoagland solution with Mo concentrations of 0 and 1 μM before simulation of drought stress. In -Mo+SNP treatment, wheat seedlings were detached from -Mo treatment and then pretreated with 100 μM SNP for 6 h during simulated drought stress. In +Mo+W and +Mo+PTIO treatments, wheat seedlings were detached from +Mo treatment and then pretreated with 100 μM tungsten (W, Mo-enzymes inhibitors) or 200 μM 2-phenyl-4,4,5,5-tetramethylimidazoline-3-oxide-1-oxyl (PTIO, NO scavenger) for 4 h, respectively, before simulation of drought stress. In +Mo+W+SNP treatment, wheat seedlings were detached from +Mo treatment and firstly pretreated with 100 μM tungsten (W, Mo-enzymes inhibitors) for 4 h, and then added with 100 μM SNP for 6 h during simulated drought stress, and each treatment was replicated four times, each replicate including 3–4 plants. After 6 h of simulation of drought stress, the detached leaves were sampled and immersed in liquid nitrogen immediately, and then stored at -80°C for further analysis.

### Analysis of Mo and Chlorophyll Contents

The Mo contents of wheat leaves were determined by oscillographic polarograph according to Sun et al. (2009). The chlorophyll contents of wheat leaves were measured using the method of Wu et al. (2014).

### Analysis of Antioxidant Enzyme Activities

Frozen wheat leaves were homogenized in 5 mL extraction buffer containing 50 mM sodium phosphate (pH 7.8), and then centrifuged at 12000 *g* for 20 min. The supernatant was used for determination of the activities of superoxide dismutase (SOD), peroxidase (POD), catalase (CAT), ascorbate peroxidase (APX) using previously described methods (Wu et al., 2014; Wu S. et al., 2015).

### Analysis of Reactive Oxygen Species

Hydrogen peroxide (H_2_O_2_) was assayed following the method of Wu Z. et al. (2015). Fresh wheat leaves were sampled and immediately homogenized in 0.1% (w/v) cold trichloroacetic acid (TCA), and the homogenate was centrifuged at 12000 *g* for 20 min at 4°C. A reaction mixture containing 0.5 mL of supernatant, 0.5 mL 100 mM potassium phosphate buffer (pH 6.8), 2 mL 1 M potassium iodide (KI), and 0.1% TCA was regarded as the blank. The mixture was incubated for 1 h in darkness, and then the absorbance was determined at 390 nm.

Superoxide anion (O_2_^–^) staining was performed using the method of Deng et al. (2015) with minor modifications. The leaf tips (5 cm) of the second fully expanded leaves were detached, and then approximately 2 cm leaf tip was cut. The remaining leaf segments (approximately 3 cm) were applied to staining. The superoxide anion was visually detected using 0.5 mg mL^-1^ nitro blue tetrazolium (NBT) solution for 8 h, and then decolorized in boiling ethanol.

### Analysis of Membrane Damage

Malondialdehyde (MDA) contents were determined using the method of Wu Z. et al. (2015). Fresh wheat leaves were sampled and immediately homogenized in 0.1% (w/v) cold TCA, and the homogenate was centrifuged at 12000 *g* for 20 min at 4°C. The reaction mixture contained 0.5 mL of supernatant, 2.5 mL 0.5% thiobarbituric acid (TBA) solution (dissolved in 20% TCA). The reaction mixture was boiled for 30 min, and then rapidly cooled and centrifuged at 12000 *g* for 5 min. The difference between the absorbance values at 532 and 600 nm with an extinction coefficient of 155 mM cm^-1^ was applied to calculate the MDA contents.

### Analysis of NR Activities

The NR activity assay followed the method of Sun et al. (2015) with some modifications. Frozen wheat leaves were homogenized in 4 mL 25 mM sodium phosphate (pH 8.7) buffer containing 10 mM cysteine, 1.3 mM ethylenediaminetetraacetic acid (EDTA), and the homogenate was centrifuged at 4000 r min^-1^ for 15 min at 4°C. The reaction mixture contained 0.4 mL of enzyme extract, 1.2 mL 0.1 M KNO_3_, and 0.4 mL 2.82 mM NADH-Na_2_. The reaction started when NADH was added, and the reaction mixture was incubated for 30 min at 25°C. The reaction mixture without NADH was used as the blank. The reaction was terminated by the addition of 1 mL 1% sulfanilamide in 3 M HCl, followed by the addition of 1 mL 0.02% N-phenyl-2-naphthylamine and reaction for 15 min. The absorbance was determined at 540 nm after centrifugation at 4000 r min^-1^ for 5 min. The calibration curve was prepared by using a standard solution of 1 μg mL^-1^ NO_2_^–^.

### Analysis of NO Content

NO contents were measured using the Griess reagent as described by Zhang et al. (2009). Frozen wheat leaves were homogenized in 4 mL 50 mM acetic acid (pH 3.6) buffer containing 4% zinc acetate, and the homogenate was centrifuged at 10000 *g* for 15 min at 4°C. Activated carbon (0.1 g) was added to the supernatant solution, which was then vibrated and filtered. The filtrate (1 mL) was treated with 1 mL 1% sulfanilamide in 3 M HCl, incubated for 10 min in darkness at room temperature, added with 1 mL 0.02% N-phenyl-2-naphthylamine and incubated for 10 min. The absorbance was determined at 540 nm after centrifugation at 4000 r min^-1^ for 5 min. The calibration curve was prepared by using a standard solution of 100 ng mL^-1^ NO_2_^–^.

Nitric oxide was visually observed by 3-amino, 4-aminomethyl-2′, 7′-difluorescein, diacetate (DAF-FM DA) as described by Luo et al. (2012). The leaf segments were incubated with10 μM DAF-FM DA dissolved in 20 mM HEPES buffer (pH 7.4) for 30 min in darkness at 37°C, and then washed with 0.2 M sodium phosphate buffer (pH 7.4) for 20 min. The images were photographed using a Nikon fluorescent inverted microscope imaging system (495 nm excitation and 515 nm emission wavelength).

### Total RNA Extraction and Quantitative RT-PCR

Frozen wheat leaves were used to evaluate the expression of antioxidant enzyme genes according to the method of Wu Z. et al. (2015). The total RNA was dissolved in 30 μL DEPC⋅H2O, and quantified with a NanoDrop 2000 UV-VIS spectrophotometer (Thermo, United States). The RNA quality was accessed by agarose gel, and then stored at -80°C. The qualified RNA was transcribed to cDNA with Oligo (dT) (Promega, Madison, WI, United States), M-MLVRTase (Promega, Madison, WI, United States) and dNTP, and then used a detection system of IQ^5^ Real-Time PCR (Bio-Rad, United States) to produce cDNA. The gene-specific primers, cDNA template and the SYBR Green mix (Bio-Rad, United States) were mixed in a 96-well plate for the following detection. The cycling program of Real-Time PCR was as below: 30 s denaturation at 95°C, and then 44 cycles of 10 s at 95°C, 20 s at annealing temperature of the primers (**Table 1**), followed by 30 s at 72°C. The primers of *TaSOD* and *TaAPX* were obtained from Li et al. (2013); the primer of *TaCAT* was obtained from Luna et al. (2005). The Actin gene primer was designed by OligoArchitecxt^TM^ Online^1^. The detailed information of the primers is available in **Table 1**. The relative expression levels of antioxidant enzyme genes were calculated as described by Pfaffl (2001).

**Table 1:** Sequences of primers used for RT-PCR.

Genes	Primer sequence 5′ to 3′	Annealing temperature (°C)	Amplification efficiency (%)	Genebank accession no.
TaSOD	F TTGTAGGTCGCTGGTTTC R CCAAGTTCACGGTTCATAG	56.4	91.7	U69536.1
TaCAT	F ACTACGACGGGCTCATG R GGAGCTGAGACGGCTTC	46	100	E16461
TaAPX	F GACGGCTGAATGGTTGAA R AATGCCTCCTGGTCCTCT	56.4	91	AF387739.1
Actin	F ACTGGGATGACATGGGGAA R ACCGCTGGCATACAAGGAC	58	95	AB181991.1
β-tubulin	F CATGCTATCCCTCGTCTCGACCT R CGCACTTCATGATGGAGTTGTAT	55	90	–

### Statistical Analysis

Statistical analyses of data were performed by SPSS 19.0 software using Duncan multiple range comparison. Graphs were plotted using Sigmaplot.

## Results

### Mo Enhanced Antioxidant Defense in Winter Wheat under Polyethylene Glycol 6000-Simulated Drought Stress (PSD)

**FIGURE 1:**
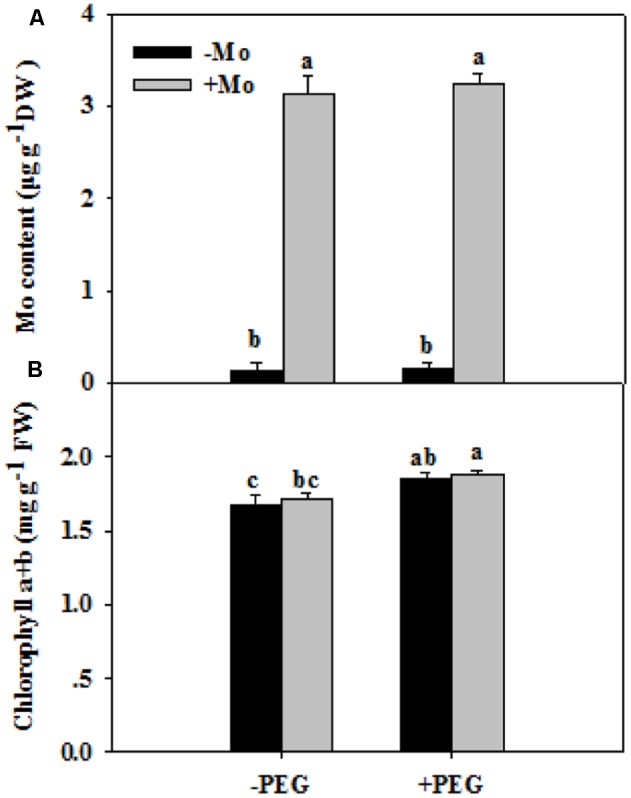
Effects of molybdenum (Mo) application on Mo contents **(A)** and chlorophyll contents **(B)** in wheat leaves at 0 and 24 h of polyethylene glycol 6000-simulated drought stress (PSD). –Mo and +Mo treatments represent wheat grown in modified Hoagland solution with 0 and 1 μM Mo, respectively. –PEG and +PEG represent 0 and 24 h of polyethylene glycol 6000 (PEG) stress, respectively. Different letters indicate significant differences between treatments by the Duncan-test (*p* < 0.05, *n* = 4).

**FIGURE 2:**
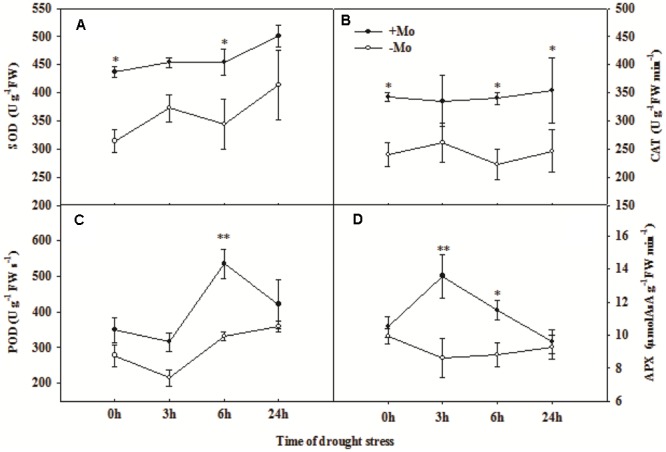
Effects of molybdenum (Mo) application on superoxide dismutase (SOD; **A**), catalase (CAT; **B**), peroxidase (POD; **C**), and ascorbate peroxidase (APX; **D**) in wheat leaves under polyethylene glycol 6000-simulated drought stress (PSD). –Mo and +Mo treatments represent wheat grown in modified Hoagland solution with 0 and 1 μM Mo, respectively. 0, 3, 6, and 24 h represent 0, 3, 6, and 24 h of polyethylene glycol 6000 (PEG) stress, respectively. Asterisk (^∗^) indicates significant difference between –Mo and +Mo treatments by ANOVA followed by Duncan-test multiple comparisons (^∗^*P* < 0.05, ^∗∗^*P* < 0.01; *n* = 3).

To assess whether Mo induces antioxidant defense in wheat leaves under PSD, the activities of antioxidant enzymes, and levels of H_2_O_2_, O_2_^–^ and MDA were determined. First, we measured the Mo and chlorophyll contents in wheat leaves at 0 and 24 h of PSD. Mo content in wheat leaves was significantly increased by Mo application (**Figure 1A**). There is no difference in chlorophyll a+b contents between -Mo and +Mo treatments (**Figure 1B**), suggesting that no Mo deficiency symptom was observed in -Mo-treated wheat. Under PSD, the activity of SOD and CAT was consistently increased, and that of POD and APX initially showed an increasing trend, followed by a decrease at 6 h and 3 h. However, the activities of SOD (0 and 6 h), CAT (0, 6, and 24 h), POD (6 h) and APX (3 and 6 h) were significantly increased by Mo application (**Figure 2**). During the whole period of PSD, the average increases of SOD, CAT, POD and APX activities in wheat leaves due to Mo application were 28.40, 41.27, 36.94 and 24.46%, respectively (**Figure 2**).

**FIGURE 3:**
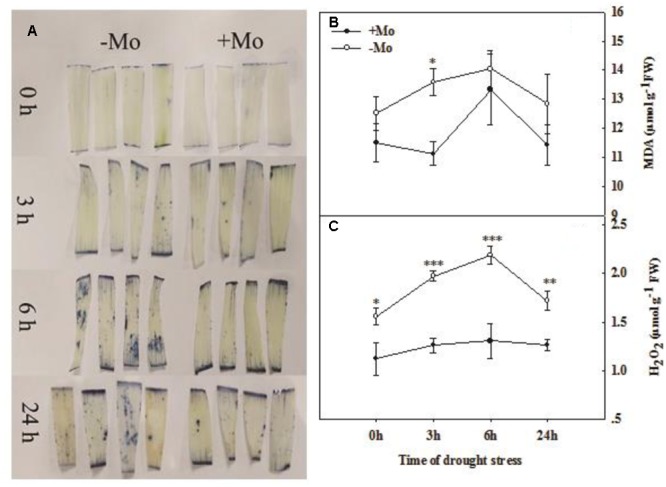
Effects of molybdenum (Mo) application on superoxide anion (O_2_^–^; **A**), malondialdehyde (MDA; **B**), and hydrogen peroxide (H_2_O_2_; **C**) in wheat leaves under polyethylene glycol 6000-simulated drought stress (PSD). –Mo and +Mo treatments represent wheat grown in modified Hoagland solution with 0 and 1 μM Mo. 0, 3, 6, and 24 h represent 0, 3, 6, and 24 h of polyethylene glycol 6000 (PEG) stress, respectively. Asterisk (^∗^) indicates significant difference between –Mo and +Mo treatments by ANOVA followed by Duncan-test multiple comparisons (^∗^*P* < 0.05, ^∗∗^*P* < 0.01, ^∗∗∗^*P* < 0.001, *n* = 4).

The O_2_^–^ was visually determined by NBT staining. The results indicated that there was an increasing trend in O_2_^–^ accumulation with prolonged PSD. The O_2_^–^ accumulation was decreased by Mo application at 3, 6, and 24 h of PSD in wheat leaves (**Figure 3A**). The MDA content in wheat leaves was decreased by Mo application and a significant difference was observed at 3 h of PSD (**Figure 3B**). The highest contents of MDA and H_2_O_2_ were observed at 6 h of PSD, and the MDA and H_2_O_2_ levels were decreased from 6 to 24 h of PSD. However, the H_2_O_2_ content was dramatically decreased by Mo application (**Figure 3C**). These results suggested that the antioxidant defense ability was improved due to the activation of antioxidant enzyme activities by Mo under PSD.

### Mo Increased NO Production through Mo-Enzymes

**FIGURE 4:**
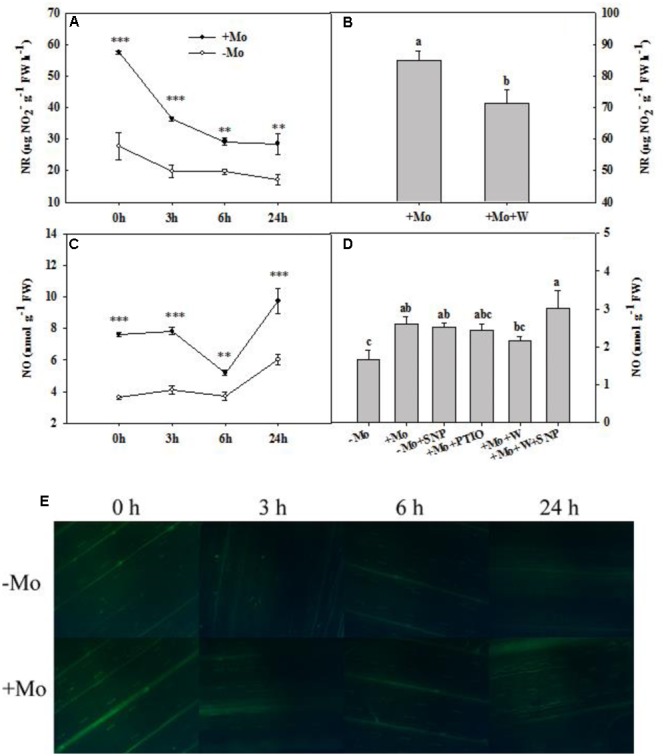
Nitrate reductase (NR) activity and nitric oxide (NO) content. Effects of molybdenum (Mo) application on NR activity **(A)** and NO production **(C,E)** in wheat leaves under polyethylene glycol 6000-simulated drought stress (PSD). –Mo and +Mo treatments represent wheat grown in modified Hoagland solution with 0 and 1 μM Mo, respectively. 0, 3, 6, and 24 h represent 0, 3, 6, and 24 h of polyethylene glycol 6000 (PEG) stress, respectively. Bar = 100 μm. Asterisk (^∗^) indicates significant difference between –Mo and +Mo treatments by ANOVA followed by Duncan-test multiple comparisons (^∗∗^*P* < 0.01, ^∗∗∗^*P* < 0.001, *n* = 4). **(B)** Effects of 100 μM tungsten (W, Mo-enzymes inhibitors) on NR activity in wheat leaves. The 30-day-old seedlings grown in +Mo Hoagland solutions were detached and placed in distilled water for 2 h to eliminate potential wound stress and firstly pretreated with/without 100 μM W for 4 h, and then exposed to 10% polyethylene glycol 6000 (PEG) for 6 h. **(D)** Effects of Mo, 100 μM W, 200 μM 2-phenyl-4,4,5,5-tetramethylimidazoline-3-oxide-1-oxyl (PTIO, NO scavenger) and 100 μM sodium nitroprusside (SNP, NO donor) on NO production in wheat leaves. The 30-day-old seedlings grown in –Mo and +Mo Hoagland solutions were detached and placed in distilled water for 2 h to eliminate potential wound stress and firstly pretreated with/without 200 μM PTIO or 100 μM W for 4 h, and then exposed to 10% polyethylene glycol 6000 (PEG) with/without 100 μM SNP for 6 h. Seedlings cultivated with –Mo and +Mo solutions were regarded as the control. Different letters indicate significant differences between treatments by the Duncan-test (*p* < 0.05, *n* = 4).

During the whole period of PSD, the NR activity of wheat leaves was decreased with/without Mo application. However, +Mo treated wheat leaves showed significantly higher NR activity than -Mo treated wheat leaves (**Figure 4A**). The NO production showed a fluctuating trend with the prolongation of PSD, and Mo application significantly enhanced the NO production of wheat leaves under PSD (**Figure 4C**). The DAF-FM DA experiment further confirmed these results (**Figure 4E**).

To verify the role of Mo in NO production, wheat leaves were exposed to PSD for 6 h in a complementary experiment. Compared with -Mo treatment, +Mo treatment and -Mo+SNP treatment substantially increased NO production. Compared with +Mo treatment, +Mo+PTIO treatment and +Mo+W treatment decreased NO production by 6.92 and 16.92%, respectively, and subsequently, there was a remarkable increase of NO production with the addition of SNP (+Mo+W+SNP) (**Figure 4D**). Compared with +Mo treatment, pretreatment with inhibitors of Mo-enzymes (100 μM W) dramatically inhibited NR activity in wheat leaves at 6 h of PSD (**Figure 4B**). These results revealed that Mo might enhance NO production by regulating NR.

### NO Mediated Mo-Induced Antioxidant Defense through Mo-Enzymes in Winter Wheat under PSD

**FIGURE 5:**
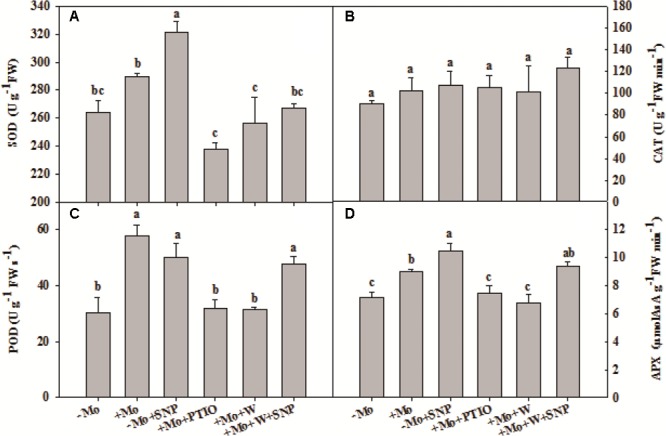
Effects of molybdenum (Mo), 100 μM tungsten (W, Mo-enzymes inhibitors), 200 μM 2-phenyl-4,4,5,5-tetramethylimidazoline-3-oxide-1-oxyl (PTIO, NO scavenger), and 100 μM sodium nitroprusside (SNP, NO donor) on activities of antioxidant enzymes in wheat leaves. The 30-day-old seedlings grown in -Mo and +Mo Hoagland solutions were detached and placed in distilled water for 2 h to eliminate potential wound stress and firstly pretreated with/without 200 μM PTIO or 100 μM W for 4 h, and then exposed to 10% polyethylene glycol 6000 (PEG) with/without 100 μM SNP for 6 h. Seedlings cultivated with -Mo and +Mo solutions were regarded as the control. Different letters indicate significant differences between treatments by the Duncan-test (*p* < 0.05, *n* = 4).

**FIGURE 6:**
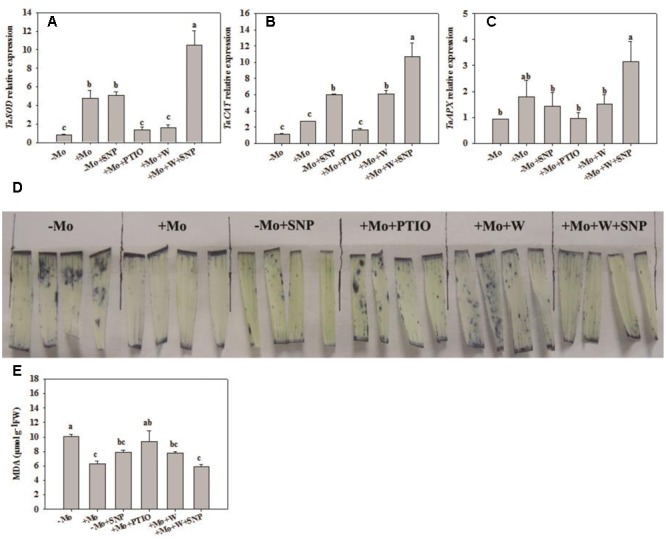
Effects of molybdenum (Mo), 100 μM tungsten (W, Mo-enzymes inhibitors), 200 μM 2-phenyl-4,4,5,5-tetramethylimidazoline-3-oxide-1-oxyl (PTIO, NO scavenger), and 100 μM sodium nitroprusside (SNP, NO donor) on transcripts of antioxidant enzymes, superoxide anion (O_2_^–^) accumulations and malonaldehyde (MDA) contents in wheat leaves. The 30-day-old seedlings grown in -Mo and +Mo Hoagland solutions were detached and placed in distilled water for 2 h to eliminate potential wound stress and firstly pretreated with/without 200 μM PTIO or 100 μM W for 4 h, and then exposed to 10% polyethylene glycol 6000 (PEG) with/without 100 μM SNP for 6 h. Seedlings cultivated with -Mo and +Mo solutions were regarded as the control. Different letters indicate significant differences between treatments by the Duncan-test (*p* < 0.05, *n* = 3).

To assess whether NO is involved in the antioxidant defense induced by Mo application, wheat leaves were pretreated with PTIO (a scavenger of NO) and W (an inhibitor of NR), and then exposed to 10% PEG with SNP for 6 h. Compared with -Mo treatment, +Mo treatment and -Mo+SNP treatment conspicuously promoted SOD, POD and APX activities. Compared with +Mo treatment, +Mo+PTIO and +Mo+W treatments substantially inhibited SOD, POD and APX activities; and subsequently, POD and APX activities were remarkably increased due to the application of SNP (+Mo+W+SNP) (**Figures 5A,C,D**). Moreover, the expression levels of *TaSOD, TaCAT*, and *TaAPX* were generally higher in Mo-treated wheat leaves than in -Mo treated leaves, with significant difference being observed in *TaSOD* (**Figures 6A–C**). Increase in the expression of *TaSOD, TaCAT*, and *TaAPX* was observed in -Mo+SNP treatment, compared with -Mo treatment. +Mo+PTIO and +Mo+W treatments decreased *TaSOD* and *TaAPX* expression relative to +Mo treatment, and the application of SNP (-Mo+W+SNP) sharply induced the expression of *TaSOD, TaCAT*, and *TaAPX* in wheat leaves under PSD (**Figures 6A–C**). Combining these physiological and molecular results, it could be speculated that NO signaling confers Mo-induced antioxidant defense through the regulation of NR, and SOD may play a more important role in the induction of antioxidant defense than other enzymes.

To further investigate the effects of NO on Mo-induced antioxidant defense, we determined the O_2_^–^ accumulation caused by water stress and content of MDA, a product of lipid peroxidation caused by ROS. Compared with -Mo treatment, +Mo treatment and -Mo +SNP treatment dramatically reduced the accumulation of O_2_^–^ in wheat leaves. Compared with +Mo treatment, +Mo+PTIO and +Mo+W treatments substantially enhanced O_2_^–^ accumulation (**Figure 6D**). However, there was a significant decrease in O_2_^–^ accumulation due to the addition of SNP in +Mo+W treated wheat leaves (**Figure 6D**). Moreover, +Mo treatment and -Mo +SNP treatment resulted in a significantly lower MDA content than -Mo treatment. +Mo+PTIO and +Mo+W led to higher MDA contents compared with +Mo treatment. However, there was a decrease of MDA due to the addition of SNP in +Mo+W treatment (**Figure 6E**). Taken together, these results indicated that NO mediates Mo-induced antioxidant defense through the regulation of NR.

## Discussion

Oxidative stress caused by ROS due to water deficit results in oxidative damage to plant protein, membrane lipid and DNA. However, antioxidant defense systems, which mainly involve the activities of antioxidant enzymes or other antioxidants, can protect plants from oxidative damage (Gill and Tuteja, 2010). Generally, oxidative damage is evaluated by MDA, a lipid peroxidation product resulting from ROS (Wu et al., 2014). In the present study, with the prolongation of PSD, oxidative damage in wheat leaves was aggravated by ROS resulting from PSD (**Figure 3**). However, oxidative damage indicated by MDA was alleviated due to the elimination of ROS by Mo application (**Figure 3**), which can be ascribed to the enhancement of antioxidant enzyme activities (**Figure 2**). The protection of plants by antioxidant defense systems against oxidative damage was triggered by antioxidant enzymes including SOD, CAT, APX and POD (Wu et al., 2014; Wu S. et al., 2015). Generally, ROS is scavenged by antioxidant enzymes. For example, SOD catalyzes O_2_^–^ into H_2_O_2_, and then H_2_O_2_ is disintegrated by CAT and APX (Wu S. et al., 2015; Wu Z. et al., 2015). Our previous studies have suggested that oxidative damage is relieved by the improvement of antioxidant defense ability by Mo in plants under cold, drought and salt stress (Sun et al., 2006; Zhang et al., 2012; Wu et al., 2014). However, the mechanisms by which Mo improves antioxidant defense ability remain to be investigated.

Nitric oxide is a signaling molecule mainly produced by NR and NOS (Sun C. et al., 2014). However, a recent study suggested that the Mo-enzymes NR and ARC could form a dual system of NR-ARC to induce NO production under nitrate condition in *Chlamydomonas* (Chamizo-Ampudia et al., 2016). Although W is not a specific inhibitor of NR, it is widely used as an inhibitor in most studies of NR-induced NO production. Since W is not a specific inhibitor of NR, it may inhibit other Mo-enzymes, such as ARC proteins, which are also involved in NO production in *Chlamydomonas* (Chamizo-Ampudia et al., 2016). However, it remains unclear whether W inhibits the ARC proteins in higher plants, particularly in wheat. In this work, 100 μM W inhibited NR activity (**Figure 4B**), but 5 mM W increased NR activity in wheat (Data not shown), indicating that different W concentrations may have different effects on Mo-enzyme activities. The result that 5 mM W increased NR activity may be supported by a previous study, in which W was suggested to induce NO production (Negi et al., 2010). Adamakis et al. (2012) and Xiong et al. (2012) reviewed that different W concentrations have different effects, and the same W concentrations also have distinct effects in different plants. More importantly, the ARC proteins still have not been identified in wheat so far. It will be interesting and significant to identify the ARC proteins and investigate the effects of Mo on ARC proteins in wheat in future studies. Besides, Chamizo-Ampudia et al. (2017) reviewed that the Mo-enzymes aldehyde oxidase (AO), sulfite oxidase (SO) and xanthine dehydrogenase (XDH) are also involved in the NO production *in vitro.* However, to our best knowledge, there are no researches confirm that AO, SO and XDH induce NO production in plants. Thus, the following discussion is focused on the well-known pathway through which NO is produced by NR in higher plants.

Nitrate reductase-dependent NO production is involved in Al tolerance (Sun C. et al., 2014), copper tolerance (Hu et al., 2014), freezing tolerance (Zhao et al., 2009), and virus tolerance (Jian et al., 2015), whereas NOS-dependent NO production is related to salt and drought tolerance (Zhao and Zhang, 2004; Zhou et al., 2005). In this study, Mo application dramatically enhanced NO content and increased NR activity in wheat leaves under PSD (**Figures 4A,C,E**), indicating that Mo may participate in NR-dependent NO production. This result is supported by the results that 100 μM W strongly inhibited NR activity and decreased NO production, while 100 μM SNP substantially restored the NO production (**Figures 4B,D**). Mo application by the methods of seed priming and foliar spray increased NR activity in wheat grass under salt stress, and application of W in the same way inhibited NR activity (Babenko et al., 2015). NO oscillation occurring in plants has been reported to facilitate the resistance against metal toxicity (Cu and Al) by alleviating oxidative damage and increasing antioxidant gene transcripts (González et al., 2012; Sun C. et al., 2014). NO oscillation in +Mo treatment was more pronounced than that in -Mo treatment (**Figures 4C,E**), which may confer drought tolerance by alleviating oxidative damages in wheat leaves. In addition, an early burst of NO is involved in the improvement of resistance to Al stress and immune response in wheat (Guo et al., 2004; Sun C. et al., 2014), and both the early NO burst and secondary NO production increase the pathogen tolerance (Floryszak Wieczorek et al., 2007). However, in this study, a burst of NO production was observed at 24 h of PSD, which may also play a key role in Mo-induced antioxidant defense in wheat leaves. It is worth noting that NR activity was not increased simultaneously with NO burst at 24 h of PSD. Thus, it can be speculated that NOS may be involved in Mo-induced NO production. This is the first report about the Mo-induced NO production at least partially via NR in wheat leaves under PSD.

Nitrate reductase-dependent NO production is related to oxidative stress tolerance in bean roots (Wang et al., 2010) and wheat roots (Sun C. et al., 2014) under Al stress, whereas NOS-dependent NO production is involved in the improvement of oxidative stress tolerance in maize, transgenic tobacco and *Stylosanthes guianensis* under drought stress (Zhou et al., 2005; Zhang et al., 2007, 2009; Sang et al., 2008a). Since other Mo-enzymes are also likely to be involved in NO production, we herein propose a possible pathway by which Mo improves antioxidant defense ability: Mo–Mo-enzymes–NO–antioxidant defense pathway. The present findings indicate that Mo induces NO production at least partially by NR (**Figure 4**), which sequentially increases antioxidant enzyme activities, and contributes to the decrease of oxidative damage caused by ROS accumulation (**Figures 2, 3**). These results also reveal that NO plays a key role in Mo-induced increase of antioxidant enzyme activities, which subsequently prevents drought-induced oxidative damage. This finding is further demonstrated by the result that -Mo+SNP treatment increased NO content (**Figure 4D**), and subsequently alleviated oxidative damages by increasing the transcripts and activities of antioxidant enzymes (**Figures 5, 6**). On the other hand, +Mo+W treatment and +Mo+PTIO treatment resulted in the decrease of NO production and subsequently the increase of oxidative damages due to the decrease in the transcripts and activities of antioxidant enzymes (**Figures 5, 6**). However, the increase of oxidative damages was inhibited by SNP. These findings suggest that NR-dependent NO production participates in Mo-induced antioxidant defense in wheat leaves. Taken together, the results strongly indicate that NO production, which is at least partially induced by NR, is regulated by Mo and is involved in the Mo-induced antioxidant defense in wheat leaves under PSD.

However, the regulatory network of NO signal is extremely complex in plant cells. Little is known about the upstream and downstream NO signals in the regulation of Mo-induced antioxidant defense under PSD. It has been reported that NO functions as a secondary signal in ABA-induced physiological responses and stress tolerance (Zhang et al., 2007; Lu et al., 2014). Moreover, Mo increases ABA biosynthesis by regulating AO (Sun et al., 2009). Thus, it will be interesting to verify the upstream and downstream NO signals in future studies. MAPK and CCaMK are downstream signal of NO in the induction of oxidative tolerance in maize (Zhang et al., 2007; Ma et al., 2012). However, *TaCCaMK* is a negative regulator for ABA signaling, which may participate in abiotic stress responses in wheat (Yang et al., 2011). This finding implies that NO may directly regulate Mo-induced oxidative tolerance in wheat. Taken together, results from this work clearly demonstrate that NO signal plays a significant role in Mo-induced tolerance of oxidative damages in wheat under PSD.

## Author Contributions

XS, CH, and SW designed the experiment. SW and SX performed the experiment. SW and QT analyzed the data. SW and XS wrote the manuscript.

## Conflict of Interest Statement

The authors declare that the research was conducted in the absence of any commercial or financial relationships that could be construed as a potential conflict of interest.

## Footnotes

1 http://www.oligoarchitect.com
